# Expression of Extracellular Vesicle PIWI-Interacting RNAs Throughout hiPSC-Cardiomyocyte Differentiation

**DOI:** 10.3389/fphys.2022.926528

**Published:** 2022-06-16

**Authors:** Ana F. Louro, Nikolaus Virgolini, Marta A. Paiva, Inês A. Isidro, Paula M. Alves, Patrícia Gomes-Alves, Margarida Serra

**Affiliations:** ^1^ iBET, Instituto de Biologia Experimental e Tecnológica, Oeiras, Portugal; ^2^ Instituto de Tecnologia Química e Biológica Aónio Xavier, Universidade Nova de Lisboa, Oeiras, Portugal

**Keywords:** extracellular vesicles, hiPSC-cardiomyocyte, piRNA, Akt, small RNA-seq

## Abstract

Extracellular Vesicles (EV) play a critical role in the regulation of regenerative processes in wounded tissues by mediating cell-to-cell communication. Multiple RNA species have been identified in EV, although their function still lacks understanding. We previously characterized the miRNA content of EV secreted over hiPSC-cardiomyocyte differentiation and found a distinct miRNA expression in hiPSC-EV driving its *in vitro* bioactivity. In this work, we investigated the piRNA profiles of EV derived from key stages of the hiPSC-CM differentiation and maturation, i.e., from hiPSC (hiPSC-EV), cardiac progenitors (CPC-EV), immature (CMi-EV), and mature (CMm-EV) cardiomyocytes, demonstrating that EV-piRNA expression differs greatly from the miRNA profiles we previously identified. Only four piRNA were significantly deregulated in EV, one in hiPSC-EV, and three in CPC-EV, as determined by differential expression analysis on small RNA-seq data. Our results provide a valuable source of information for further studies aiming at defining the role of piRNA in the bioactivity and therapeutic potential of EV.

## Introduction

The secretion of membrane vesicles is an evolutionarily conserved biological phenomenon observed across organisms, that mediates intricate processes of cellular regulation and coordination essential for survival (Deatheragea and Cooksona, 2012). These extracellular vesicles (EV) are nano and micro-sized particles without a functional nucleus, that transport a cargo of complex biomolecules involved in intra- and intercellular communication (Van Niel et al., 2018). Although EV can carry an assortment of genetic material, including both DNA and RNA, they are mostly enriched in small non-coding RNAs (ncRNA) (Nolte-’t Hoen et al., 2012). ncRNAs mediate evolutionarily conserved silencing pathways by regulating mRNA stability or translation, or by targeting epigenetic modifications to specific regions of the genome, and are involved in the control of gene regulatory programs (Statello et al., 2020) in development and disease (Li et al., 2019; Rayford et al., 2021). Multiple ncRNA species have been identified in EV, including microRNA (miRNA), ribosomal RNA (rRNA), transfer RNA (tRNA), long non-coding RNA (lncRNA), PIWI-interacting RNA (piRNA), small nuclear RNA (snRNA), and small nucleolar RNA (snoRNA) (Nolte-’t Hoen et al., 2012; Kaur et al., 2018). Though there is no consensus on their relative percentage, several studies suggest miRNA accounts for 40%–70% of ncRNA in EV (Huang et al., 2013; Yuan et al., 2016). In opposition, other authors argue that most protocols for small RNA library construction use adaptor ligations that favor miRNA, thus introducing a significant bias in biotype identification (Raabe et al., 2014; Wei et al., 2017). Regardless of the reason, miRNAs are the most extensively studied ncRNA, and only a few studies focusing on other EV ncRNA molecules have been performed so far.

Advanced analytical techniques such as deep sequencing have allowed the identification of other RNA biotypes in EV samples, with piRNAs emerging as potential regulators and/or biomarkers of disease (Li et al., 2019; Abramowicz and Story, 2020; Zeng et al., 2021). piRNAs are a class of single-stranded ncRNAs associated with the PIWI (P element-induced wimpy testis) subset members of the Argonaut family of proteins, with 24–34 nucleotides (nt) in length, that direct the silencing of transposable elements (TEs), thereby conserving genome integrity (Wu et al., 2020). In humans, there are 4 PIWI homologs, PIWIL1 (HIWI1), PIWIL2 (HILI), PIWIL3 (HIWI3), and PIWIL4 (HIWI2) (Wu et al., 2020).

Thousands of piRNA have been identified across the mammalian genome. They mostly arise from TEs but can also originate from long intergenic non-coding RNAs (lincRNAs), noncoding, nonrepetitive genomic elements (piRNA clusters), and in rarer cases, from protein-coding genes (Sarkar et al., 2014). PIWI proteins and piRNA were initially thought to be restricted to animal germline (Girard et al., 2006) and stem cells, and their presence in somatic tissues is not well characterized, with controversy remaining around their existence and function. Several studies on piRNA support its existence in the soma (Yan et al., 2011; Rajasethupathy et al., 2012; Ross et al., 2014), with tissue-specific expression patterns, and have demonstrated its functional role in epigenetic reprogramming, regulation of transcription, translation, and development (Wu et al., 2020). Nevertheless, as of yet, few studies have shown that interfering with somatic piRNA or with the genes involved in its biogenesis induces majorly altered phenotypes (Jones et al., 2016), in opposition to what occurs in germ lines in which mutations in PIWI-family genes causes significant defects in germline development, namely infertility, across species (Schmidt et al., 1999; Carmell et al., 2007).

Groundwork on the identification of piRNA cargo of EV and its part in intercellular communication has been emerging but it is rather preliminary in comparison with the extensive portrayal of EV-miRNA.

In a previous study, we characterized the bioactivity and miRNome of human induced pluripotent stem cells (hiPSC)- and hiPSC-cardiomyocytes (hiPSC-CM)-derived EV and found distinct EV potency and miRNA expression patterns during differentiation (Louro et al., 2022). Moreover, we corroborated the upregulation of stemness maintenance miRNA clusters in hiPSC-EV and showed that hiPSC-EV targeted the PI3K/AKT pathway, a crucial network in heart physiopathology, by mediating PTEN suppression and increasing AKT activity. Remarkably, it has been reported that piRNAs may also influence the PI3K/AKT signaling pathway (Rajan et al., 2014; Vella et al., 2016), and could thus have an important role in angiogenesis, proliferation and survival (Rajan et al., 2014). In this context, identification of piRNAs in EV secreted by hiPSC, cardiac progenitor cells (CPC) and hiPSC-CM could provide further insights into the mechanisms underlying EV bioactivity and uncover new therapeutic approaches for cardiovascular diseases.

## Methods

### hiPSC Culture and Differentiation Into Cardiomyocytes

hiPSC (IMR90-4 line; WiCell) were expanded on coated plates (Matrigel hESC-Qualified Matrix, Corning), in feeder-free, animal component-free, TeSR-E8 medium (STEMCELLTechnologies), in a humidified atmosphere at 37°C and 5% CO_2_. Differentiation was initiated when cells reached 80%–90% confluency following a previously published protocol for Wnt/ß-catenin temporal modulation (Lian et al., 2013; Correia et al., 2016). On differentiation day 0, expansion medium (TeSR-E8™) was replaced by differentiation medium (RPMI 1640 (Gibco^®^, ThermoFisher Scientific) with B27 without insulin (ThermoFisher Scientific) (RPMI + B27-I), supplemented with CHIR99021 (12 μM) (Tocris Bioscience), Activin A (80 ng ml^−1^) (Tebu-bio) and ascorbic acid (50 μg ml^−1^) (Sigma Aldrich). After 24 h (day 1), the medium was replaced by differentiation medium supplemented with IWR1 (5 μM) (Selleckchem) and ascorbic acid (50 μg ml^−1^). At day 3 (72 h after differentiation induction), medium was exchanged for differentiation medium supplemented with IWR-1 (5 μM). At day 6, medium was replaced by maintenance medium [RPMI 1640 supplemented with B27 with insulin (RPMI + B27)]. hiPSC-CM aggregates were generated between days 7–8 of differentiation by forced aggregation in AggreWell™ 400 plates (STEMCELL Technologies), according to the protocol described by (Correia et al., 2018) CM aggregates were harvested 48 h after aggregation and transferred to an orbital suspension culture system, at an agitation rate of 90 rpm. Maintenance medium was changed every other day thereafter until day 15 of culture. From day 15 onwards, RPMI + B27 medium was replaced by metabolic maturation medium, comprising RPMI 1640 without glucose (MP biomedicals, ThermoFisher Scientific) supplemented with B27, 1 mM of glutamine (Sigma-Aldrich), 10 mM of D(+)-galactose (Sigma-Aldrich), 100 μM of oleic acid (Sigma-Aldrich) and 50 μM of palmitic acid (Sigma-Aldrich). Maturation medium was changed 3 times per week thereafter. hiPSC-CM aggregates were cultured in these conditions for additional 20 days, reaching a total of 35 days of culture since the start of the differentiation process (Correia et al., 2017; Louro et al., 2022).

### Extracellular Vesicles Isolation and Characterization

EV were isolated from equal volumes of conditioned culture medium (240 ml) by differential ultracentrifugation and density gradient ultracentrifugation, and characterized at days 0 (expansion medium), 6 (differentiation medium), 15 (maintenance medium), and 35 (maturation medium) of culture. Briefly, conditioned medium was centrifuged twice at low speeds (10 min at 300 g, followed by a second centrifugation for 10 min at 2000 g; rotor A-4-81, 5810 R centrifuge, Eppendorf) to remove major contaminants. The resulting supernatant was filtered through 0.45 µm filter units (Nalgene™ Rapid-Flow™, Thermo Fisher Scientific) and ultracentrifuged in 30 ml conical open-top polyallomer tubes (Beckman Coulter) for 3 h using a XL-100 ultracentrifuge (SW 28 rotor, Beckman-Coulter) at 110,000 g_max_ to create an EV pellet. An OptiPrep™ density gradient (ODG, Axis Xield Diagnostics) was prepared as reported by (Louro et al., 2022). The gradient was prepared by consecutive layering on a 16.8 ml open-top polyallomer tube (Beckman Coulter) 4 ml of 40% (containing the EV sample), 4 ml of 20%, 4 ml of 10%, 3.5 ml of 5% iodixanol solutions, and 1 ml of PBS. The gradient was centrifuged at 4 °C for 18 h at 110,000 g_max_ (SW 28.1 rotor, Beckman Coulter). After centrifugation, 16 fractions of 1 ml were collected from top (fraction 1) to bottom (fraction 16), and divided into six samples by pooling fractions 1–4, 5–7, 8–9, 10–12, and 13–16. Pooled gradient fractions 8–9 (EV containing fractions) were concentrated to 300 µL using Amicon^®^ Ultra-2 ml 10 KDa filter units (Merck Millipore), aliquoted, and stored at −80°C until further characterization.

### RNA Isolation

Total RNA was isolated from EV samples (N = 3 EV preparations from 3 independent cultures of four cell populations, in a total of 12 EV samples) using Norgen Biotek Exosomal RNA Isolation Kit (Cat.58000), according to the manufacturer’s instructions.

### Library Preparation and Small RNA Sequencing

Next-generation sequencing of small RNAs was performed by Norgen Biotek Corp, as described in (Louro et al., 2022). Briefly, small RNA library preparation was performed by PCR amplification using Norgen Biotek Small RNA Library Prep Kit (Cat. 63600). Library quality control, including estimation of library size and concentration, was achieved using Bioanalyzer. Sequencing was performed using Illumina’s NextSeq 500 sequencing platform.

### Sequence Alignment

FASTQ files were processed using the exceRpt small RNA-seq pipeline version 4.6.2, available on the Genboree Workbench (http://genboree.org/theCommons/projects/exrna-tools-may2014/wiki/Small_RNA-seq_Pipeline) by Norgen Biotek Corp. Processed reads were aligned to the human reference genome (hg38) and annotated using the corresponding reference transcriptome. piRNA sequences were aligned and annotated using the RNAdb reference database (Pang et al., 2005, 2007).

### Length Frequency and Small RNA Biotype Distribution Analysis

Read lengths and biotype count files generated by the small RNA-seq pipeline on the Genboree Workbench were used for length frequency and biotype distribution analysis.

### Analysis of Differentially Expressed piRNAs

Data filtering, normalization and differential expression (DE) analysis were conducted using the EdgeR (Robinson et al., 2009) package (version 3.14) within the R environment (version 4.1). Data was filtered based on a minimum of 5 counts per million and normalized using the trimmed mean of M-values (TMM) method (Robinson and Oshlack, 2010). DE analysis was performed using an exact test for differences between each two groups. False discovery rate was adjusted through the Benjamini-Hochberg procedure (Benjamini and Hochberg, 1995). DE was considered significant for |log_2_ fold change| ≥ 1, with a *p*-value ≤ 0.05 and FDR ≤ 0.05. Principal component analysis (PCA) was performed using the DESeq2 (v1.34.0) R package (Love et al., 2014). Soft clustering was implemented with R package Mfuzz (v2.42.0) (Futschik et al., 2005; Kumar and Futschik, 2007). R packages ComplexHeatmap (v2.10.0) and EnhancedVolcano (v1.12.0) were used to plot heatmaps and volcano plots, correspondingly.

### RT-qPCR Validation of Differentially Expressed piRNAs

Differentially expressed piRNAs were validated by reverse transcription quantitative real time polymerase chain reaction (RT-qPCR). Briefly, RNA samples were reverse transcribed with TaqMan™ MicroRNA Reverse Transcription Kit (Applied Biosystems™, Thermo Fisher Scientific) and Custom Taqman™ Small RNA Assays (Applied Biosystems™, ThermoFisher Scientific), according to the manufacturer’s protocol. RT-qPCR was performed using the TaqMan™ Fast Advanced Master Mix (Applied Biosystems™, ThermoFisher Scientific) on a LightCycler 480 Instrument II 384-well block (Roche) in the following cycles: UNG activation at 50°C for 2 min; polymerase activation at 95°C for 20 s; 40 cycles of amplification with denaturation at 95°C for 3 s, and annealing/extension at 60°C for 30 s. The Cycle threshold (Ct) was determined using the LightCycler 480 software (Roche). Stem-loop primers used for cDNA synthesis and RT-qPCR are available in Supplementary Table S1. Two endogenous controls (hsa-miR-103a-3p and U6) were selected based on a comprehensive assessment of expression stability in our small RNA-seq data. Relative changes were analyzed using the ∆∆Ct method (Livak and Schmittgen, 2001) (CPC-EV vs. hiPSC-EV, CMi-EV vs. CPC-EV).

### piRNA Alignment Prediction

piRNA alignment coordinates with the human reference genome as well as associated genes and transposable elements overlapping the alignment positions were found through piRNA database (www.pirnadb.org; version 1.8.0; human reference genome h38) and piRNAQuest (http://bicresources.jcbose.ac.in/zhumur/pirnaquest/index.html; version 1.0; human reference genome h19).

### Statistical Analysis

All subsequent statistical analysis performed in this manuscript were done using GraphPad Prism v9. Significance was tested by a one sample *t*-test against a hypothetical value of 1, taken as the expression of the corresponding control [*p < 0.05, **p < 0.01, ***p < 0.001, ****p < 0.0001, n.s. (*p* > 0.05)]. Results are plotted as mean ± standard deviation (N = 3 biological replicates).

## Results

### piRNA Detection in Extracellular Vesicles

**FIGURE 1:**
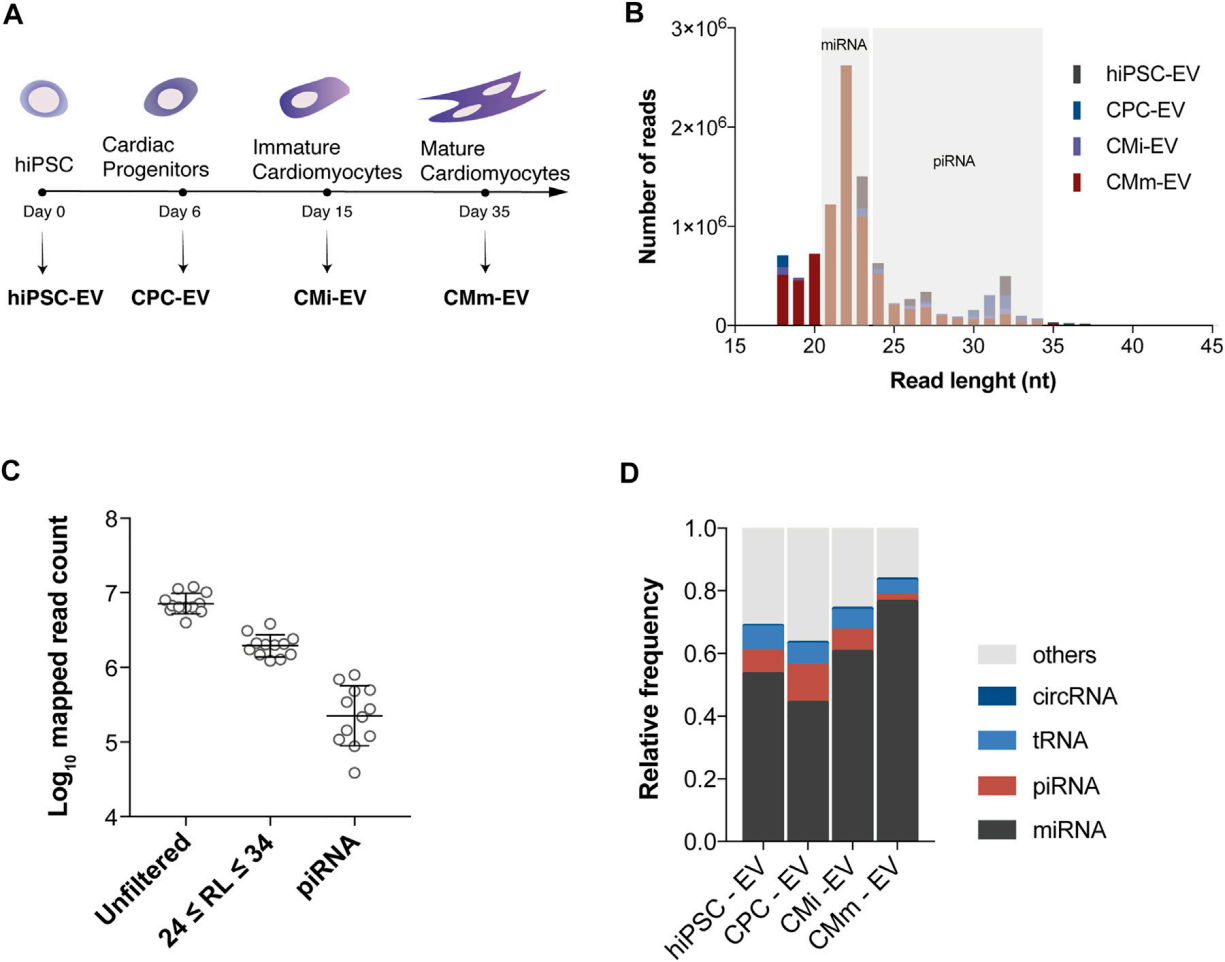
piRNA transcripts are present in extracellular vesicles. **(A)** Schematic timeline for isolation of extracellular vesicles (EV) over hiPSC-CM differentiation and maturation. EV were isolated from conditioned culture media at the hiPSC (hiPSC-EV), cardiac progenitor (CPC-EV), immature (CMi-EV), and mature cardiomyocyte (CMm-EV) stages. **(B)** Length frequency of unfiltered mapped reads (average of three biological replicates). Gray areas denote the size range for miRNAs and piRNAs. **(C)** Number of mapped reads for all samples before processing (unfiltered), after filtering by read length (RL) in the size range of piRNA, and by biotype. **(D)** Distribution of small RNA biotypes relative to reads used for alignment, for each EV population studied. Each fraction represents the mean of three replicates.

piRNA expression was analyzed by small RNA-seq in three biological replicates of EV derived from four distinct cell populations: hiPSC (hiPSC-EV), CPC (CPC-EV), immature cardiomyocytes (CMi-EV), and cardiomyocytes matured by metabolic modulation (CMm-EV), along a continuous differentiation process (Figure 1A). Analysis of length distribution on reads used for alignment, revealed the presence of three peaks, consistent for all the samples (Figure 1B). The major peak represented mature miRNA, approximately 22 nt long, while two minor ones represented piRNA, with a size range of 24–34 nt (Figure 1B). Nearly 60% of the mapped reads matched to miRNA, and the remaining reads to other non-coding RNAs. Filtering of the mapped reads based on piRNA size resulted in the removal of 65%–84% of reads (Figure 1C). After an additional filtering step based on mapped biotype for the removal of other non-coding RNAs in the same size range, only 2%–12% of the initial mapped reads remained. The experimental replicates behaved consistently throughout the filtering steps. Overall, piRNA represented the second most abundant small RNA biotype in 3 EV populations, mapping to 7.2% ± 1.1% of sequences in hiPSC-EV, 11.9% ± 1.5% in CPC-EV and 6.8% ± 1.7% in CMi-EV. In CMm-EV piRNA corresponded only to 2.1% ± 0.2% of mappings, being the third most frequent small RNA biotype (Figure 1D).

### Extracellular Vesicles piRNA Expression Profiles Along hiPSC-CM Differentiation

**FIGURE 2:**
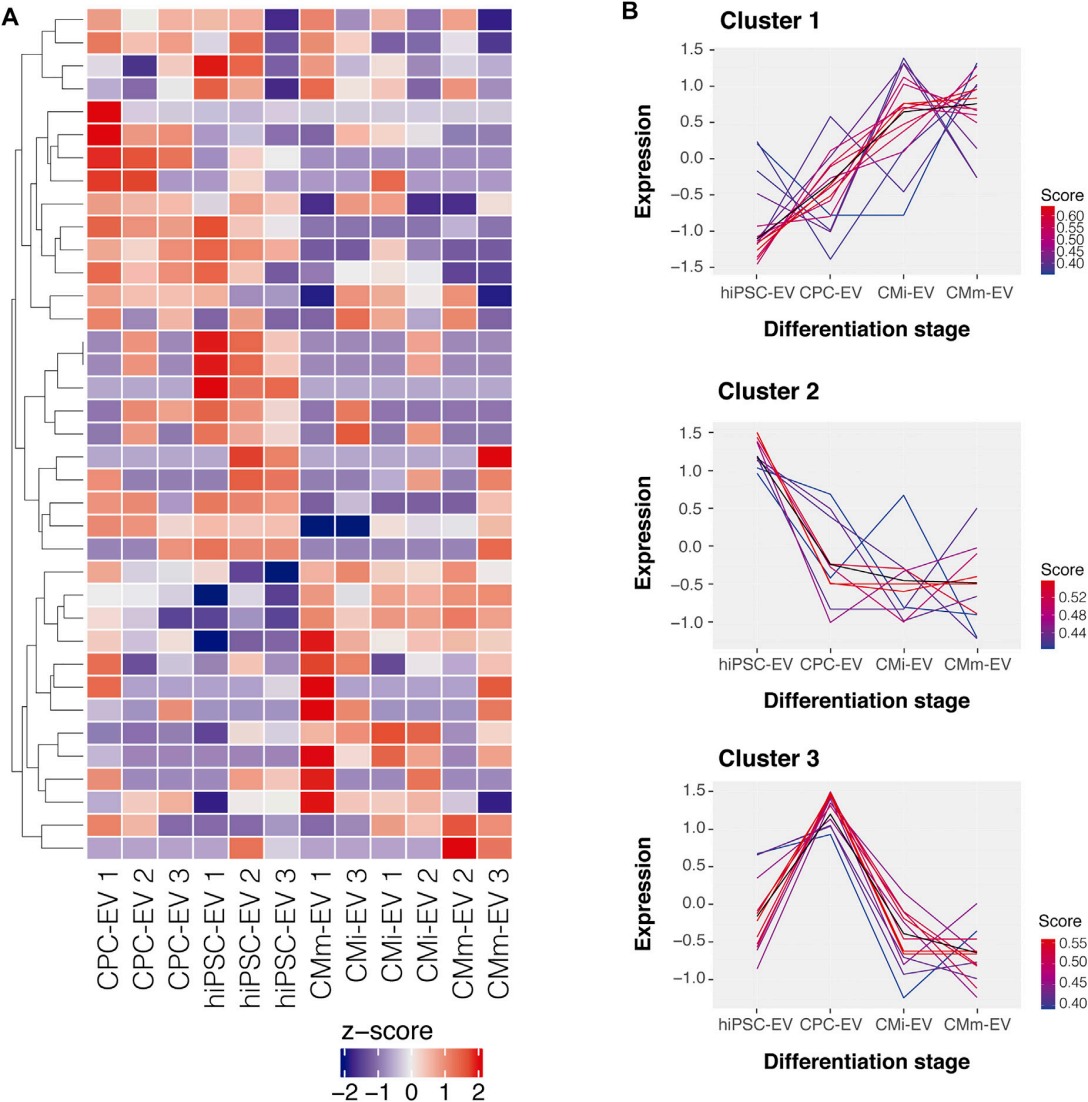
Global piRNA expression profiles during hiPSC-CM differentiation. **(A)** Heatmap representation of the global piRNA expression profiles after filtering and normalization, in three biological replicates of hiPSC-EV, CPC-EV, CMi-EV and CMm-EV, with a minimum count of 5 reads in at least one EV population. Dendrograms are based on complete-linkage hierarchical clustering and Euclidean distances. **(B)** Fuzzy plots representing the dynamic expression of 3 piRNA clusters found in EV during hiPSC-CM differentiation and maturation, obtained by soft-clustering.

We evaluated the piRNome profile of EV-derived from four cell populations. A total of 293 piRNA were identified in EV samples, and after filtering to a minimum of 5 counts per million in at least 3 samples (the smallest group size), only 37 piRNA were considered reliable for further analysis (Supplementary File S1—TMM normalized counts). The global piRNA expression differed between EV groups, with stage specific patterns emerging (Figure 2A). Fuzzy clustering was employed to analyze the dynamic expression variations throughout hiPSC-CM differentiation. In general, three broad piRNA clusters were identified (Figure 2B): one cluster showed low expression in hiPSC-EV and consistently increased throughout cardiomyocyte differentiation and further maturation; a second cluster declined upon hiPSC commitment to the cardiac fate and remained stable from CPC-EV to CMm-EV; and the third cluster was only upregulated in CPC-EV. piRNA representing each of these clusters can be found in Supplementary Table S2.

### Differentially Expressed piRNA in hiPSC-CM Derived Extracellular Vesicles

**FIGURE 3:**
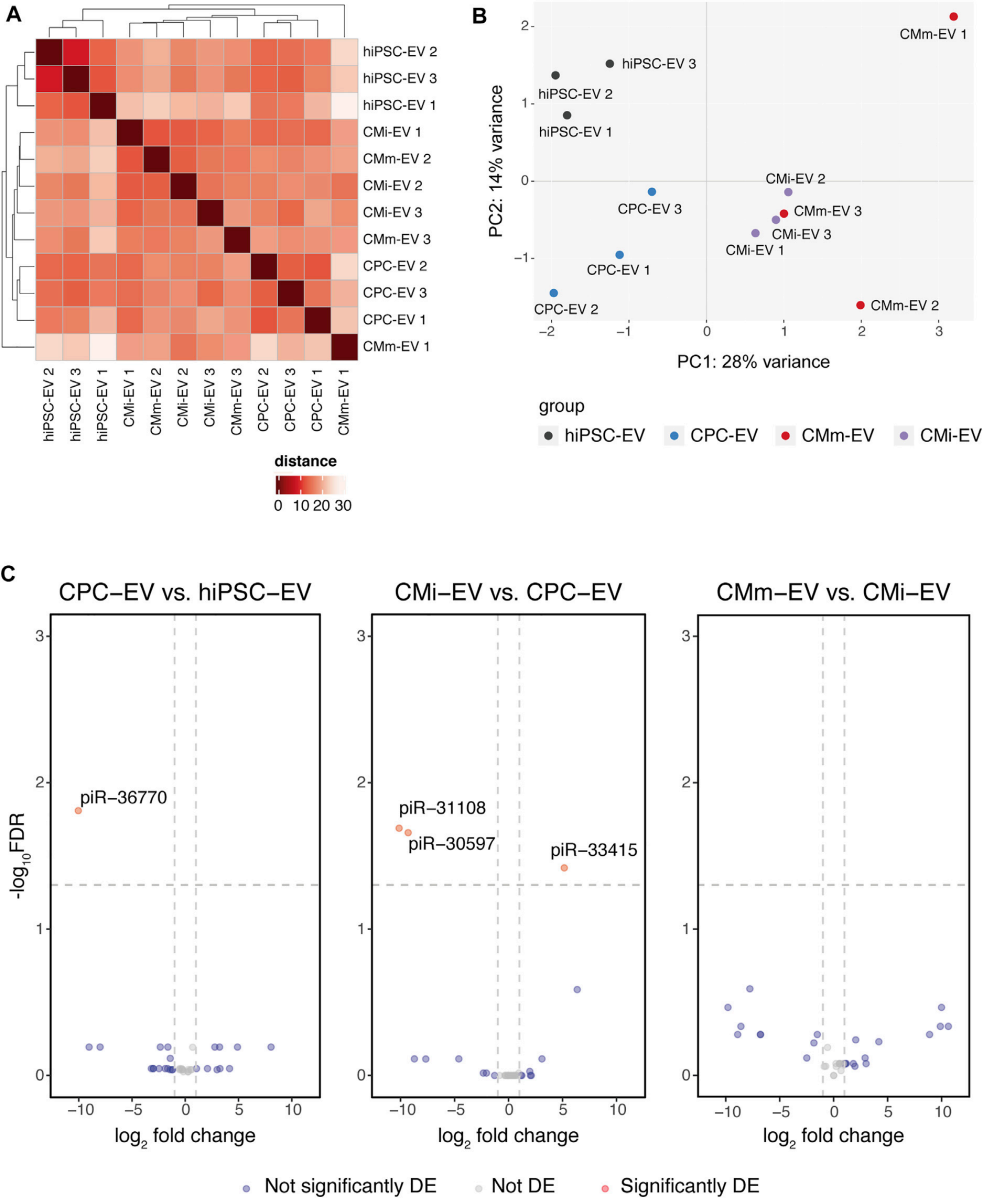
piRNA differential expression in hiPSC-CM extracellular vesicles (EV). **(A)** Heatmap with hierarchical clustering of sample-to-sample distances on the matrix of log_2_ TMM-normalized piRNA expression. Darker red colours indicate a more similar expression (colour key in arbitrary units). **(B)** Principal component analysis (PCA) performed on normalized counts. Only 42% of the variance could be explained by PC1 and PC2. **(C)** Volcano plots with pairwise comparisons of significantly differentially expressed piRNA between EV populations (CPC-EV vs. hiPSC-EV, CMi-EV vs. CPC-EV, CMm-EV vs. CMi-EV), with |log_2_ fold change| ≥ 1 and −log_10_ FDR ≥1.3. DE, differentially expressed.

Hierarchical clustering on the sample-to-sample distances from normalized gene counts revealed limited differences among EV populations (Figure 3). hiPSC-EV and CPC-EV clustered together within their corresponding group, while CMi-EV and CMm-EV replicates were assorted in a third cluster, with the exception of CMm-EV replicate 1 that showed the highest difference and did not cluster with any of the other samples. This replicate was maintained, and it did not interfere with DE analysis.

These results were confirmed by PCA. Principal component 1, explaining 28% of the variation, separated hiPSC-EV and CPC-EV from CM-EV, while principal component 2, explaining 14% of the variation, distinguished hiPSC-EV from all other groups (Figure 3B).

Globally, only 4 piRNA were found to be differentially expressed (Table 1, Supplemental file 2) at a |log_2_ fold change| ≥1 and -log_10_ FDR ≥1.3. However, only piR-36770 and piR-30597 were consistently validated by RT-qPCR (Supplementary Figures S1A,B). In fact, the magnitude of the log_2_ fold change obtained by both techniques differed greatly (Supplementary Figure S1C), although the same tendency was observed for all piRNA probed.

**TABLE 1:** Differentially expressed piRNA between CPC-EV vs. hiPSC-EV and CMi-EV vs. CPC-EV obtained by small RNA-seq. FC, fold change; CPM, counts per million; FDR, false discovery rate.

piRNA ID	NCBI accession	log_2_ FC	log_2_ CPM	*p-value*	FDR
CPC-EV *vs*. hiPSC-EV					
piR-36770	DQ598704.1	−10.0488	4.9371	0.0004	0.0155
CMi-EV vs. CPC-EV					
piR-31108	DQ570996.1	−10.1405	5.0960	0.0006	0.0205
piR-30597	DQ570485.1	−9.3024	5.4073	0.0012	0.0220
piR-33415	DQ593303.1	5.1638	5.3864	0.0031	0.0382

### Extracellular Vesicles piRNA Mapping

Differentially expressed piRNA identified both by small RNA-seq and RT-qPCR were mapped to repeat elements contained in transposons using piRNAQuest. While no association between piR-30597 and these elements was found, piR-36770 mapped to numerous LTR/ERV-1 repeats in several chromosomes (Supplementary File S3).

## Discussion

RNA-carrying vesicles are released by cells into the extracellular space, allowing for the horizontal transfer of genetic material. This mechanism of paracrine communication allows the dissemination of messages that can regulate the function of target cells (Valadi et al., 2007). Several types of short ncRNA were found to be particularly enriched in these vesicles, including miRNA, tRNA, piRNA, and snoRNA. Numerous studies have highlighted the role of ncRNA in the heart (Bian et al., 2021; Martyniak et al., 2021; Bencun et al., 2022), in parallel with the growing evidence on EV as key players in cardiac diseases and heart regeneration (Adamiak and Sahoo, 2018; Santoso et al., 2020; Lima Correa et al., 2021).

Here, we performed an exploratory study into the piRNA content of EV released during the differentiation of cardiomyocytes from hiPSC. In a previous investigation, we established a method for EV isolation and characterization from conditioned culture media and uncovered the EV miRNome from these cell sources (Louro et al., 2022). The pathophysiological role of piRNA in the heart is still poorly understood and has been the focus of recent studies (Rajan et al., 2014, 2016; Zeng et al., 2021). However, very few have focused on the EV piRNome (Wang et al., 2020).

Herein, we observed that piRNA were the second most abundant small RNA species in EV from undifferentiated and early stages of hiPSC-CM differentiation (Figure 1D). Our results indicate that piRNA transcripts are present in CM-EV, thus providing evidence of the existence of somatic piRNA. Overall, piRNA were the second most abundant small RNA biotype in hiPSC-EV, reaching a peak in CPC-EV, decreasing in CMi-EV, and considerably declining in CMm-EV (Figure 1D), suggesting an association with the pluripotent state and early stages of cell differentiation, hypothesis that requires further validation.

Employing a soft clustering algorithm, we identified three different patterns of piRNA expression (Figure 2B) with similar trends to some of the ones identified by La Greca and colleagues when characterizing the piRNome of hiPSC, early mesoderm progenitor cells, and cardiomyocytes (Greca et al., 2020). The authors identified 447 piRNA transcripts, with 8 distinct patterns, of which 3 consistently decreased over differentiation, and 3 consistently increased until later maturation stages. Compared to cells, EV had a much lower abundance of piRNA (only 37 identified transcripts), a discrepancy that indicates that piRNA accumulation in EV may be limited, in comparison to miRNA.

Even though stage-specific piRNA patterns can be inferred, the four EV populations presented low variation between each other (Figures 3A,B). In fact, the hierarchical clustering showed that samples were quite similar to each other, the highest difference occurring in hiPSC-EV, which clustered independently from all other EV samples. These results suggest that in opposition to the miRNA cargo of EV, which mimics the content and allows a strict characterization of the differentiation stage of its parent cell, the EV piRNA cargo is, in general, less correlated with cell differentiation profiles. Nevertheless, differences in piRNA profiles between hiPSC-EV and CPC-EV suggest there may be a function for these piRNA in fine-tuning transcript levels during cardiac commitment, piR-36770, overexpressed in hiPSC-EV, mapped to several Human endogenous retroviral H (HERVH) elements, implying a possible role of this piRNA in the regulation of the pluripotent state. In mammals, several ERVs are known to have stage-specific expression during embryonic development, and to be crucial for stem cell self-renewal (Santoni et al., 2012; Ohnuki et al., 2014; Pontis et al., 2019). HERVH is a primate-specific ERV family and one of the most predominant ERVs in pluripotent stem cells. In fact, HERVH repeats contained in lincRNAs exhibit a stem cell-specific expression pattern (Kelley and Rinn, 2012), and are required for pluripotency maintenance (Lu et al., 2014). In hPSC, the HERVH subfamily has numerous binding sites for and recruits several pluripotency transcription factors such as NANOG, OCT4, SOX2, and KLF4 (Kunarso et al., 2010; Ohnuki et al., 2014; Wang et al., 2014; Pontis et al., 2019), crucial for reprogramming towards iPSCs and maintenance of pluripotency and self-renewal features. Moreover, RNA interference knockdown of individual HERVH-derived lncRNAs in iPSC results in loss of pluripotency (Ohnuki et al., 2014).

piR-30597 and piR-33415 had already been detected by Vella and colleagues in adult CPC obtained from right atrial biopsies, and cultured as 3D clusters, known as cardiospheres (Vella et al., 2016). piR-30597 was detected by RT-qPCR in EV samples, but no relevant interactions were found for it. To the best of our knowledge, piR-31108 had not been described in the cardiac context. piRNA sequences are stored in databases with distinct nomenclature and accession codes, a lack of consensus may introduce a bias in literature revision, as publications may address the same piRNA by a different alias. To overcome this limitation, we performed a literature search for the differentially expressed piRNA with accession codes for NCBI (https://www.ncbi.nlm.nih.gov), ENA (https://www.ebi.ac.uk/ena), piRNABank (http://pirnabank.ibab.ac.in), piRBase (www.pirnadb.org; version 1.8.0), and piRNAQuest (http://bicresources.jcbose.ac.in/zhumur/pirnaquest/) databases.

Since we had previously identified the PI3K/AKT signaling as one of the pathways involved in hiPSC-EV bioactivity, we were prompted to search for piRNA in our dataset that targeted this pathway. Lucchinetti and colleagues showed that inhibition of LINE-1 retrotransposon expression in the heart activated the PI3K/AKT signaling (Lucchinetti et al., 2006). Similar results were obtained by Vella, which showed that several piRNA upregulated in cardiospheres targeted LINE retrotransposons throughout the genome, of which LINE-1 was most targeted, and that reduction of L1 expression was accompanied by activation of the AKT pathway (Vella et al., 2016). However, none of the piRNA in our dataset mapped to LINE-1, suggesting another mechanism of action for these piRNA. This reasoning is supported by Du and colleagues who described the role of piR-31115 - also present in our dataset (Supplementary File S2)—in renal cell carcinoma progression by activation of epithelial-mesenchymal transition via the PI3K/AKT signaling, independently of L1 repression (Du et al., 2021), as this piRNA also does not map to LINE-1.

Some limitations should be considered when interpreting the results of this study. First, small RNA-seq was performed only on EV and not on their respective cell biofactories. As such we are not able to conclude about any particular enrichment of the identified piRNA in EV versus their parent cells. Second, only an EV subfraction, enriched in small-EV was analyzed. We did not study other EV subpopulations nor compared their small ncRNA content. Third, besides piRNA, total small RNA of the size range of 24–34 nt may contain contaminants of abundant endogenous ncRNAs (such as tRNAs or rRNAs), and degradation products of longer RNAs. Indeed, total small RNA-seq reads provide information of the entire profile of expressed small RNAs, but not on which Argonaute protein a sequence associates with (Iwasaki et al., 2017). Therefore, future studies should combine total small RNA-seq data with small RNA-seq information derived from small-RNAs co-immunoprecipitated with PIWI proteins, technique that as far as we know has not been reported in the literature for EV. Finally, a great number of very low count tags accounted for most of our piRNA dataset and upon filtering based on the number of reads, few piRNA were left to perform DE analysis. In turn, this resulted in few piRNA differentially expressed, which compromised pathway enrichment analysis. As such, though we identified piRNA likely involved in particular cellular transitions, we were unable to disclose the mechanisms underlying their activity.

To conclude, we identified for the first time, global piRNA signatures in EV secreted over hiPSC-CM differentiation. The data reported in this work are a valuable source of information for further studies aiming at understanding the role of piRNA in the bioactivity of EV, in order to clarify their therapeutical relevance.

## Data Availability

Publicly available datasets were analyzed in this study. This data can be found here: https://www.ncbi.nlm.nih.gov/geo/, GSE179323; https://evtrack.org, EV210151.

## Supplementary Material

Supplementary Information supplementary figures and tables.

Supplementary File S1 TMM Normalized counts for the 37 piRNA transcripts.

Supplementary File S2 Differential expression analysis between the four EV populations.

Supplementary File S3 piR-36770 mapping to human chromosomes.
