# Supplementary material for: Expression of Extracellular Vesicle PIWI-Interacting RNAs Throughout hiPSC-Cardiomyocyte Differentiation
Source: Front Physiol. 2022 Jun 16;13:926528. doi: 10.3389/fphys.2022.926528 (PMC9243413; doi:10.3389/fphys.2022.926528)
Supplement: Supplementary file 1 [file DataSheet1.zip › Supplemental Information.DOCX]

Supplementary Material

## Supplementary Figures


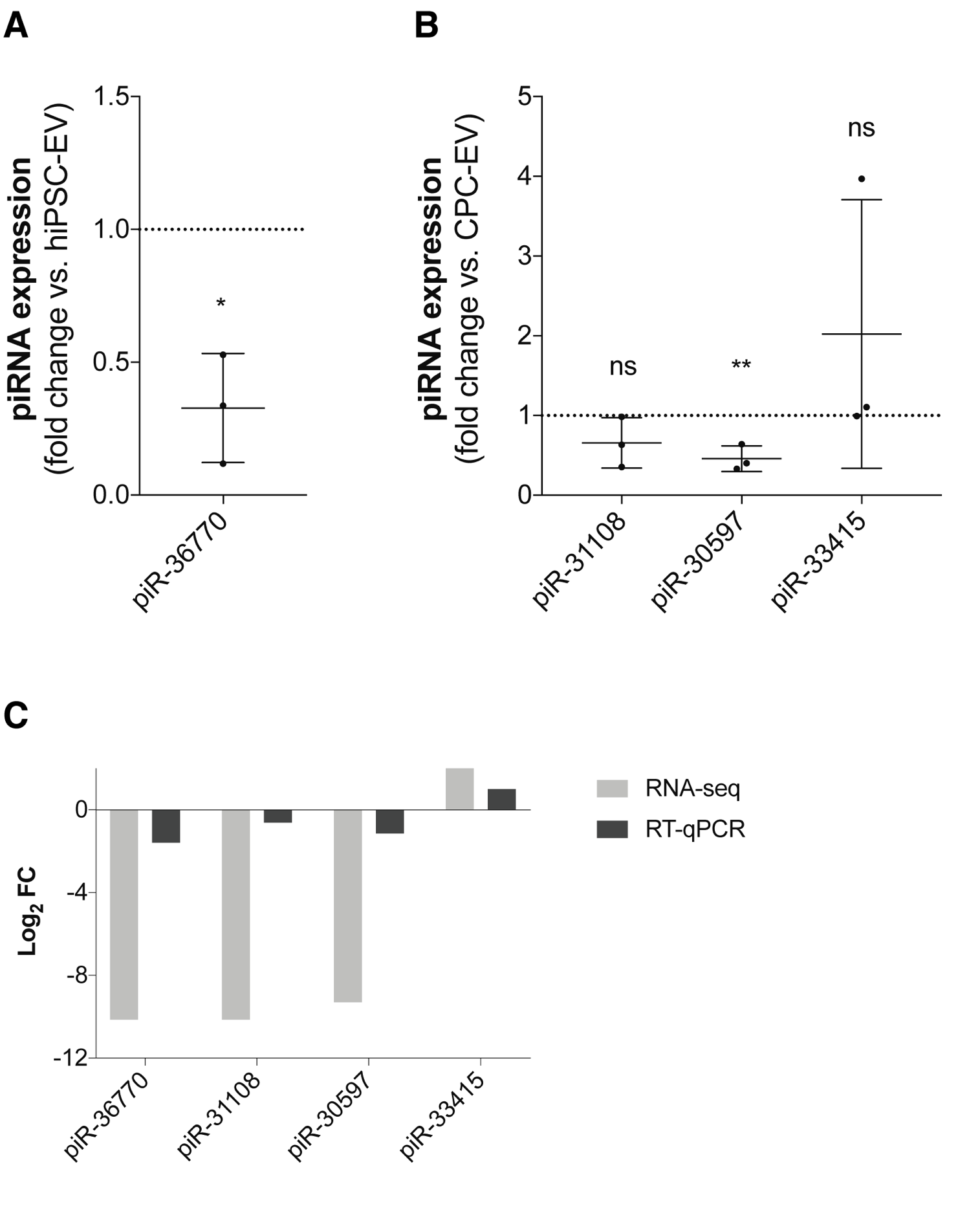


**Figure S1.** RT-qPCR validation of piRNA differentially expressed in small RNA-seq between A) CPC-EV and hiPSC-EV (piR-36770) and B) CMi-EV and CPC-EV (piR-31108, piR-30597, piR-33415). Data are mean ± SD, n=3. Relative ratios were calculated in relation to the corresponding control. Significance tested by a one-sample t-test. *p < 0.05. C) Magnitude of the log_2_ fold change obtained for piRNA identified by small RNA-seq and RT-qPCR analysis.

## Supplementary Tables

## Table S1. List of primers used for RT-qPCR validation of small RNA-seq.

| ID | Target sequence |
| --- | --- |
| hsa-miR-103a-3p | AGCAGCAUUGUACAGGGCUAUGA |
| U6 snRNA | GTGCTCGCTTCGGCAGCACATATACTAAAATTGGAACGATACAGAGAAGATTAGCATGGCCCCTGCGCAAGGATGACACGCAAATTCGTGAAGCGTTCCATATTTT |
| piR-36770 | UAAAAAGGUGGCUGGAGCCAAAGGCA |
| piR-31108 | AGCCGAGAUAGCUUCCUGAAACGUGUGAAGGA |
| piR-30597 | ACCCCUUGUGAAGCCCAAGAUCGUCAAAA |
| piR-33415 | CGCGGGUUCGAUCCCCGUACGGGCCACC |

Table S2. piRNA memberships determined by fuzzy clustering.

| **piRNA ID** | **Cluster** | **Membership** |
| --- | --- | --- |
| piR-42844 | 1 | 0.5120 |
| piR-31115 | 1 | 0.5241 |
| piR-43772 | 1 | 0.3725 |
| piR-34804 | 1 | 0.5381 |
| piR-33864 | 1 | 0.3845 |
| piR-33748 | 1 | 0.3503 |
| piR-35284 | 1 | 0.4192 |
| piR-33415 | 1 | 0.4230 |
| piR-33151 | 1 | 0.5605 |
| piR-60573 | 1 | 0.5866 |
| piR-31531 | 1 | 0.4736 |
| piR-36712 | 1 | 0.3872 |
| piR-33536 | 1 | 0.5133 |
| piR-36229 | 1 | 0.6227 |
| piR-61298 | 2 | 0.4022 |
| piR-54907 | 2 | 0.4319 |
| piR-43771 | 2 | 0.4604 |
| piR-30112 | 2 | 0.4872 |
| piR-37017 | 2 | 0.4246 |
| piR-33527 | 2 | 0.5232 |
| piR-33526 | 2 | 0.5232 |
| piR-36770 | 2 | 0.5542 |
| piR-33856 | 2 | 0.4018 |
| piR-54381 | 2 | 0.5484 |
| piR-59892 | 2 | 0.4259 |
| piR-33043 | 3 | 0.5085 |
| piR-33044 | 3 | 0.5062 |
| piR-36173 | 3 | 0.4522 |
| piR-34736 | 3 | 0.3899 |
| piR-36170 | 3 | 0.4454 |
| piR-36169 | 3 | 0.5282 |
| piR-30597 | 3 | 0.4169 |
| piR-33880 | 3 | 0.4998 |
| piR-49645 | 3 | 0.4213 |
| piR-36339 | 3 | 0.4452 |
| piR-31286 | 3 | 0.5561 |
| piR-31108 | 3 | 0.5534 |
